# Supplementary material for: Extensive spatial impacts of oyster reefs on an intertidal mudflat community via predator facilitation
Source: Commun Biol. 2022 Mar 22;5:250. doi: 10.1038/s42003-022-03192-4 (PMC8940938; doi:10.1038/s42003-022-03192-4)
Supplement: Supplementary file 2 — Supplementary Material [file 42003_2022_3192_MOESM2_ESM.pdf]

Supplementary Information to: Extensive spatial impacts of oyster reefs on an intertidal mudflat community via predator facilitation

Reddin CJ[1,2*], Decottignies P[1], Bacouillard L[3], Barillé L[1], Dubois SF[3], Echappé C[1], Gernez P[1], Jesus B[1], Méléder V[1], Nätscher P[4], Turpin V[1], Zeppilli D[5], Zwerschke N[6], Brind'Amour A[7], Cognie B[1]

Contents

Appendix S1: Supplementary Results 2
Ground truthing MPB 2
Additional MPB images 7
MPB time series 10
Sediment and organic matter 12
Macrofauna details 15
Reef epifauna details 25
Meiofauna details 26
The timescale of the impact 28
Wider situation of the reefs 29
Appendix S2: Supplementary Methods 29
Further procedural details of the reef burning 29
SEM equations and further details 32
Geostatistical details 33
Wider acknowledgements 34
Supplementary references 34

Appendix S1: Supplementary Results

Ground truthing MPB

Cell means from NDVI images were uncorrelated with ground-truthed MPB means, which is unsurprising given the high variance due to biofilm patchiness beneath the resolution of remotely-sensed images, demonstrated by the triplicate ground-truthed sample errors (Fig. S1). However, significant differences among the ground-truthed cells (ANOVA, $P \leq 0.001$) agreed that cells in the vicinity of the reef (L7, L8, L9) had significantly higher biomass than those more distant (L16 to L24; Tukey's test, $P < 0.05$). In 2014, the low biomass values from L4 to L8 were explained by the down migration of MPB cells prior to tidal submersion, which also prevented the quantification of points L1 to L3 before the end of the sampling session (Fig. S1).

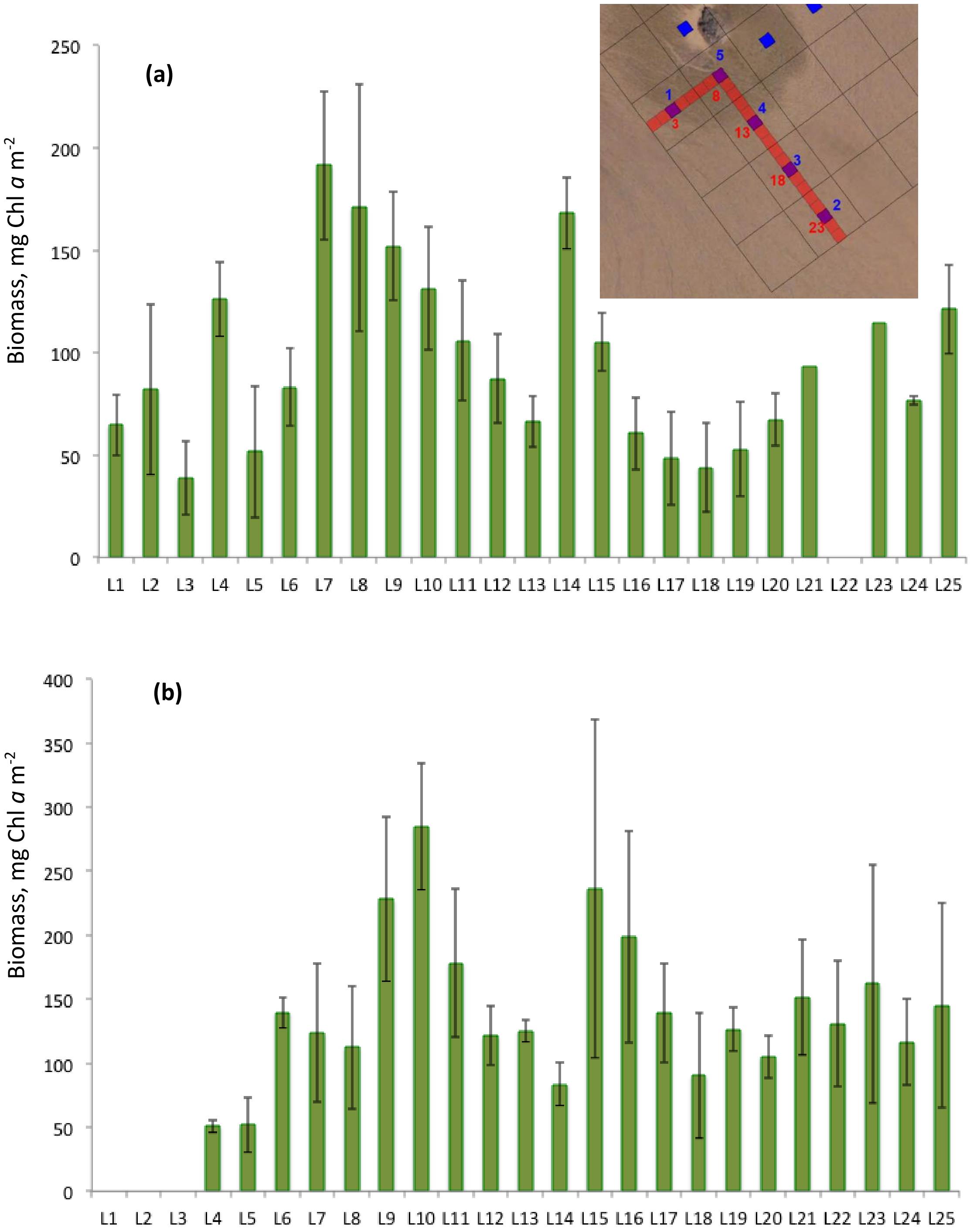

Figure S1. Ground-truthing of MPB biomass on the 'L' shape at the southern tip of the grid (inset) during (a) September 2013 and (b) October 2014. X-axis shows the station number on the 'L' (see inset and also Figure 1 in main manuscript), with L8 falling closest to the control reef. Error is SD.

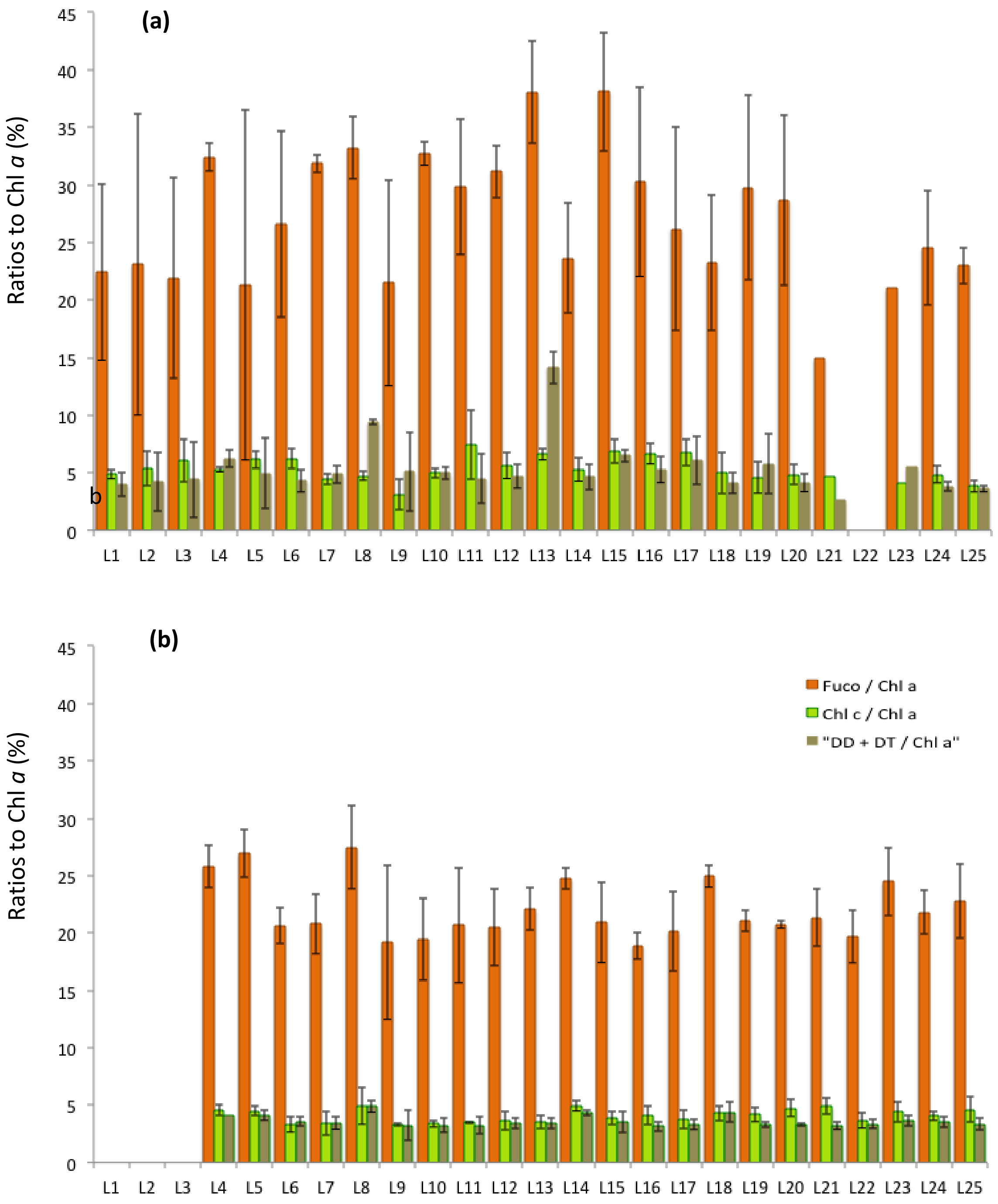

Figure S2. Ground-truthing of MPB diversity by dominant pigments relative to Chl *a*: Fuco (orange), Chl c (light green) and DD+DT (khaki). Results for (a) September 2013 and (b) October 2014. Error is SD.

Table S1. The degree of spatial variation in pigment ratios among L points (L1 to L15) from early autumn 2013 and 2014. [(1)]ANOVA tests where distributions were approximately normal; [(2)]Kruskal-Wallis tests were otherwise used.

	2013	2014
Fuco/Chl *a*	$p < 0.05^{(1)}$	$p < 0.01^{(1)}$
DD+DT/Chl *a*	$p < 0.0001^{(1)}$	$p = 0.13^{(2)}$
Chl *c*/Chl *a*	$p < 0.01^{(2)}$	$p = 0.06^{(1)}$
By-products/Chl *a*	$p < 0.0001^{(1)}$	$p < 0.0001^{(1)}$
Pheo *a*/Chl *a*	$p < 0.05^{(1)}$	$p < 0.01^{(2)}$

High concentrations of chlorophyll c and fucoxanthyll relative to chlorophyll a revealed an MPB dominance by diatoms (Fig. S2). In 2013, the ratios of fucoxanthin, Chl c and Diatoxanthin with Datioxanthin (DD+DT) showed significant spatial variation likely related to the reefs, with L9 was different (lower) from other L cells for Chl c, and L8 and L13 for DD+DT (higher by Tukey's test). Spatial variability of Chl c and DD+DT ratios were no longer significant in 2014 (Table S1). No other pigments were detected.

Breakdown products of photosynthetic pigments were in lower quantities near the reef in 2013 (Fig. S3), most likely deriving from the senescence of autochthonous diatoms (also see 1). The ratio of pheophorbid *a* did not show a relationship to the reef but did suggest that rates of grazing, such as by the mobile herbivore P. ulvae (2), rose from 2013 to 2014.

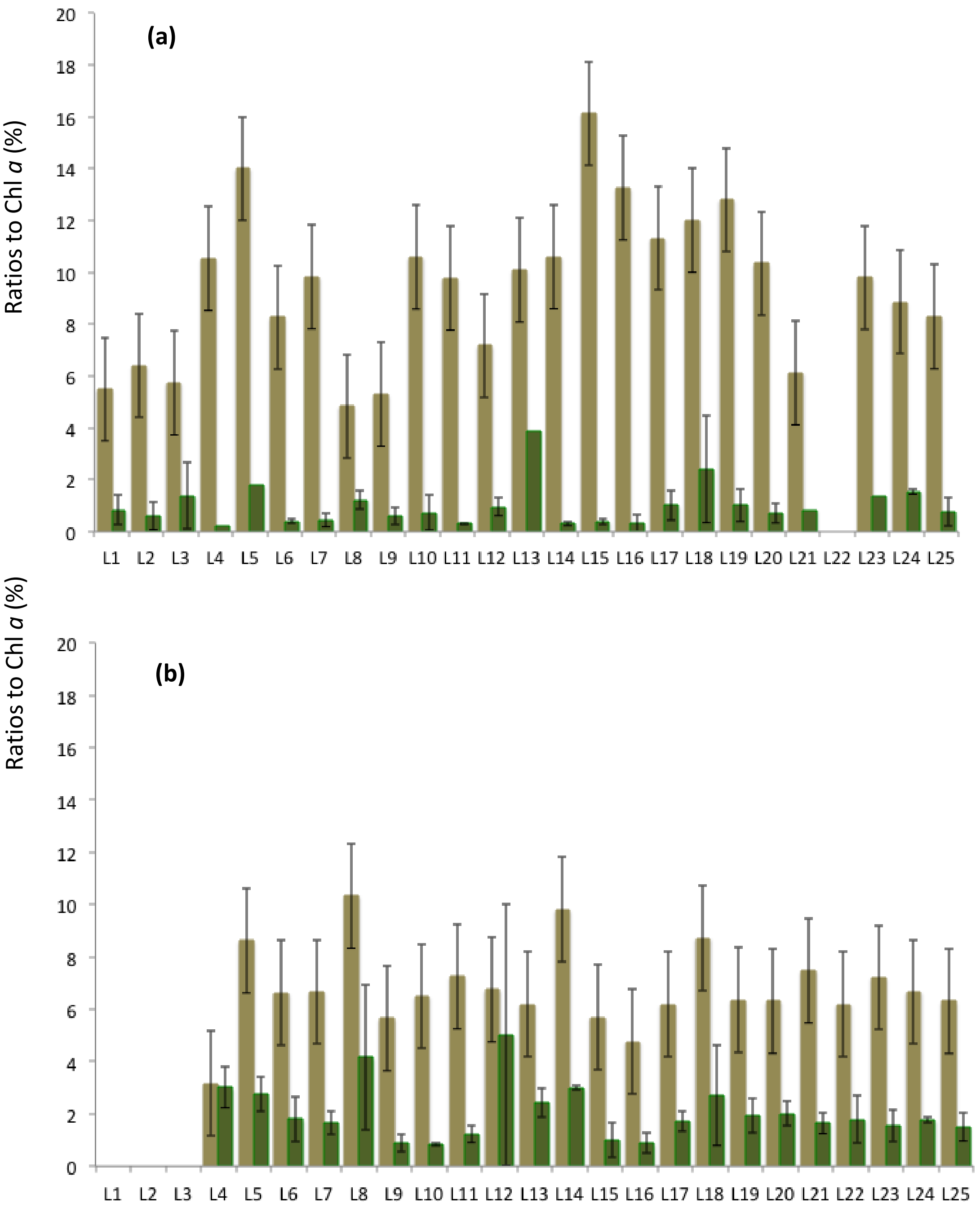

Figure S3. Ground-truthing of MPB grazing activity by by-products (khaki) and pheophorbid *a* (dark green) pigments relative to Chl *a*. Results for (a) September 2013 and (b) October 2014. Error is SD.

Additional MPB images

During March 2014, before the treatment, NDVI was as much related to distance from the large rock contained in the control reef (r = -0.17, df = 84.2, p = 0.123) and to bathymetry (r = -0.17, df = 36.4, 0.29; or non-spatial correlation with grid Y-axis, rho = 0.29, p < 0.001), as it was to distance from the reefs (NDVI vs. √ distance from either reef, r = -0.17, df = 40.7, p = 0.264; Fig. S4).

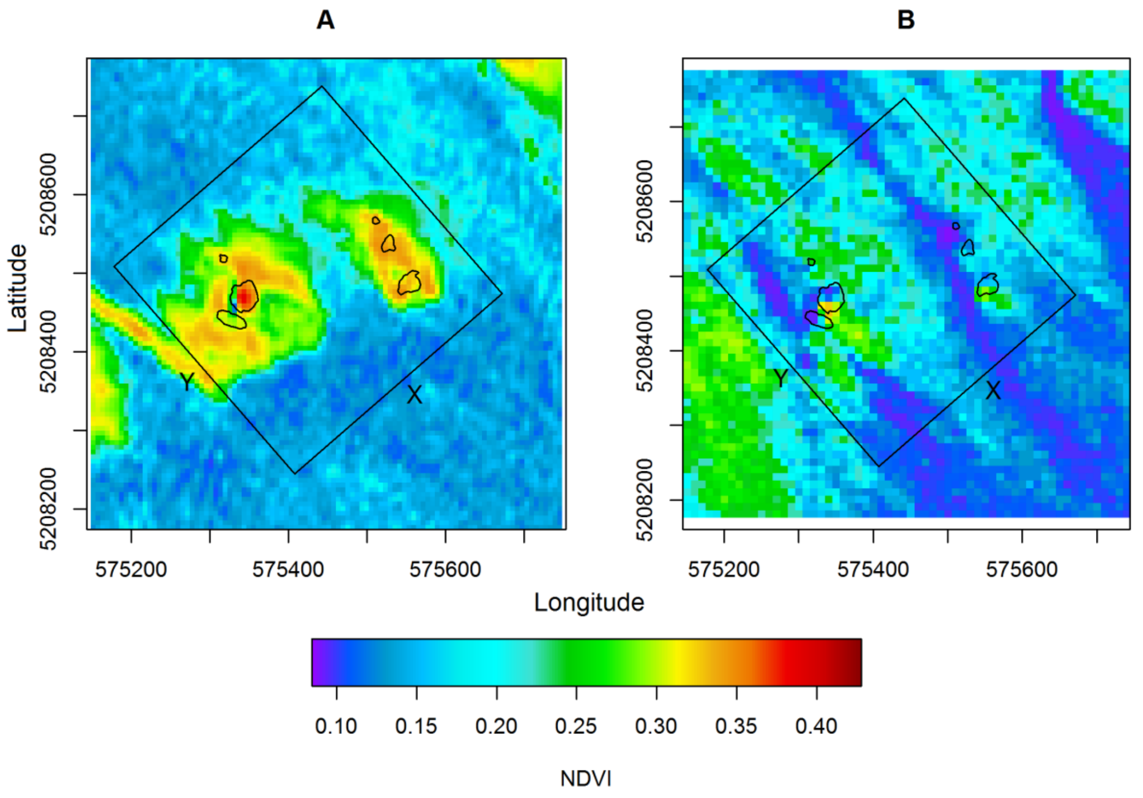

Figure S4. A comparison of NDVI images between high and low MPB growing conditions, respectively (A) September 2013 (i.e. Fig. 3) and (B) 20th March 2014 10:40 (low tide at 12:15) on the same scale. (B) is a SPOT5 image, HRG1 sensor, at 10 m resolution, with FLASH atmospheric correction and US40 (US standard, visibility 40 km) aerosol model. The black square shows extent of field-sampled grid. Black contours within this show the extent of oyster reefs.

Useful satellite images were limited by coincidence of the passing of a satellite with a sufficient imaging resolution (≤ 10 * 10 m) and optimal intertidal imaging conditions, including tidal exposure of the mudflat, daylight, a cloud-free sky and, in winter, the Sun being above an angle relative to the horizon. We chose the best available NDVI images to compared with the field sampling campaigns (Figs. 3, S4), representative of summer and winter conditions. All winter images were available only shortly after tidal emersion of the mudflat (approx. 1 hour 45 mins before peak low tide), and after the sampling campaign of that season. However, physical disturbance of the mudflat surface by the sampling campaigns is expected to be at too fine a scale to affect the satellite NDVI images. The long-term study of satellite images for this study extent in Echappé et al. (3) and Figs. S5 and S6, supports the temporal representativeness of the focal images (i.e. Fig. 3 and S4) for the seasons of summer and winter.

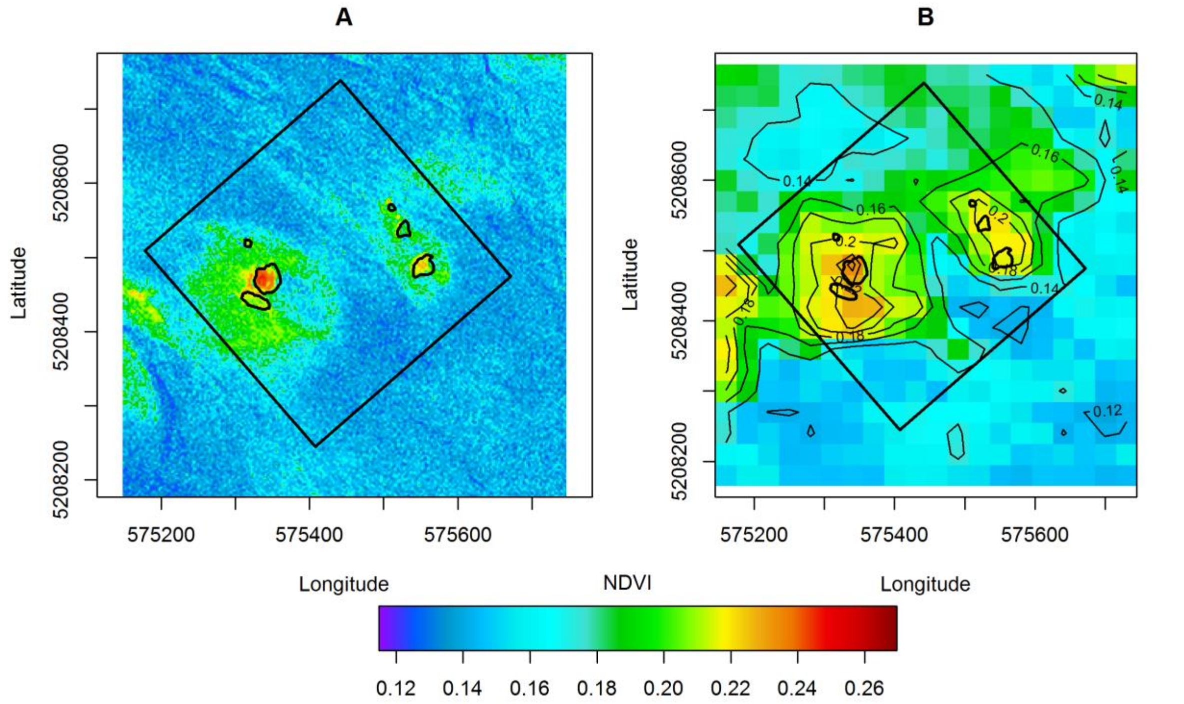

Fig. S5. Alternative summer NDVI images taken on (A) 11th July 2013 approximately 20mins prior to low tide, and (B) on 24th July 2013 11:00, approximately 10mins after low tide (10:53). (A) was taken by the Pléiades-1B satellite sensor, at 0.5m resolution, while (B) was taken by the LandSat 8 satellite, OLI sensor, at 30 m resolution, both with FLASH atmospheric correction and US40 (US standard,

visibility 40 km) aerosol model. Black square shows extent of field-sampled grid. Heavy black contours within this show the extent of oyster reefs, light black contours show NDVI patterns.

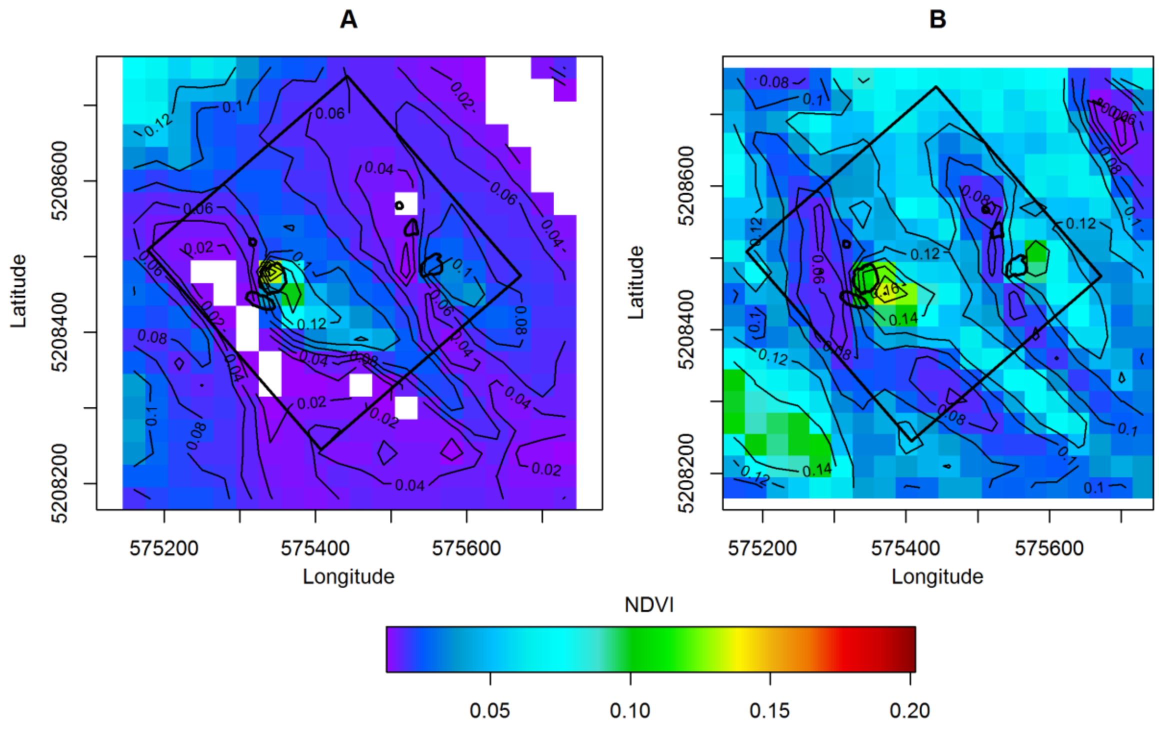

Fig. S6. Alternative winter NDVI images taken on (A) 5th March 2014 approximately 109mins prior to low tide, and (B) on 21st March 2014 approximately 113mins prior to low tide (Pornic). Images taken by the LandSat 8 satellite, OLI sensor, at 30 m resolution, with FLASH atmospheric correction and US40 (US standard, visibility 40 km) aerosol model. Black square shows extent of field-sampled grid. Heavy black contours within this show the extent of oyster reefs, light black contours show NDVI patterns.

MPB time series

The MPB response at the treatment reef was not immediate (Fig. S7). By the time of the 2014 early autumn sampling, the image closest to the sampling date, taken 83 days after the burning of the reefs, also shows an overall higher NDVI. Wider extent images suggested that this overall increase in MPB occurred across the bay. Echappe et al. (3) monitored temporal changes of the broader bay area and calibrated changes in MPB NDVI in one reef relative to the other based on long-term (25 years) data. Therefore, we can have confidence in which MPB changes recorded from 2013 to 2014 were happening across the bay and which were specific to our sampling grid (e.g. Underwood 1991). The ground-truthing values, above, also support a higher MPB biomass at 2014 sampling than at 2013 sampling, so image differences are not simply because of stage in the MPB tidal vertical migration.

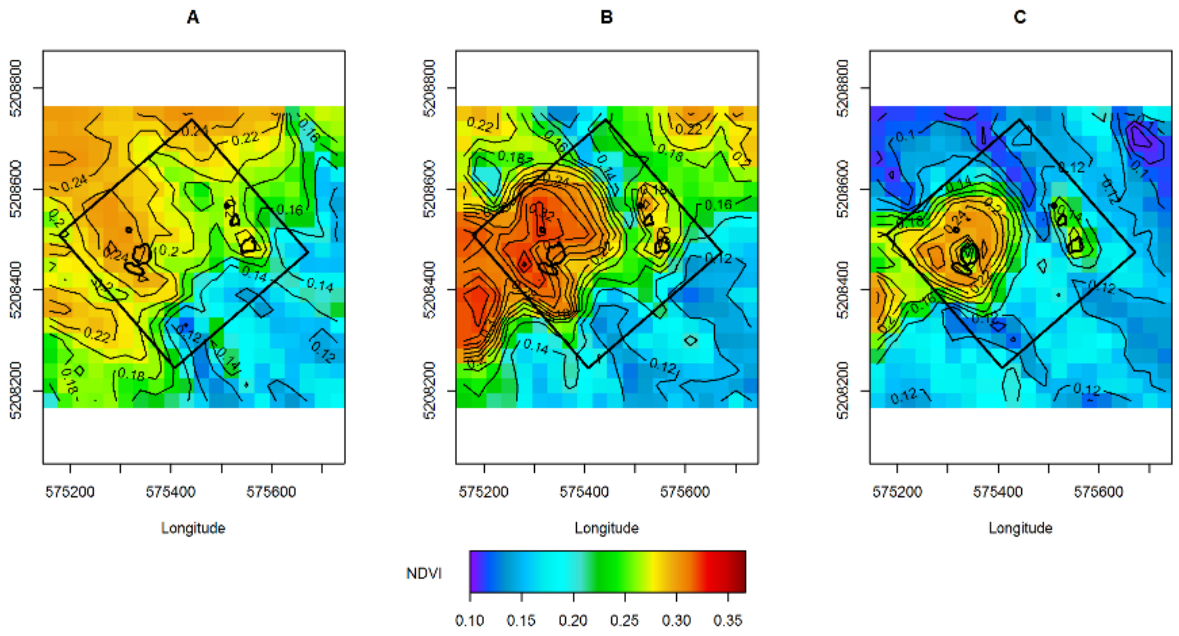

Fig. S7. Time series of NDVI images showing (A) prior to the treatment, and (B and C) after the treatment. The chronological sequence is image (A) from 17th May 2014 10:53, approximately 43mins prior to low tide (Pornic, 11:36), the treatment on 17th July 2014, image (B) taken on 28th

August 2014 10:59, approximately 25mins prior to low tide at 11:24, and image (C) taken on 29th September 2014 10:59, approximately 118mins prior to low tide at 12:57. Each image was taken by the LandSat 8 satellite, OLI sensor, at 30 m resolution, with FLASH atmospheric correction and US40 (US standard, visibility 40 km) aerosol model. Black square shows extent of field-sampled grid. Heavy black contours within this show the extent of oyster reefs, light black contours show NDVI patterns.

Sediment and organic matter

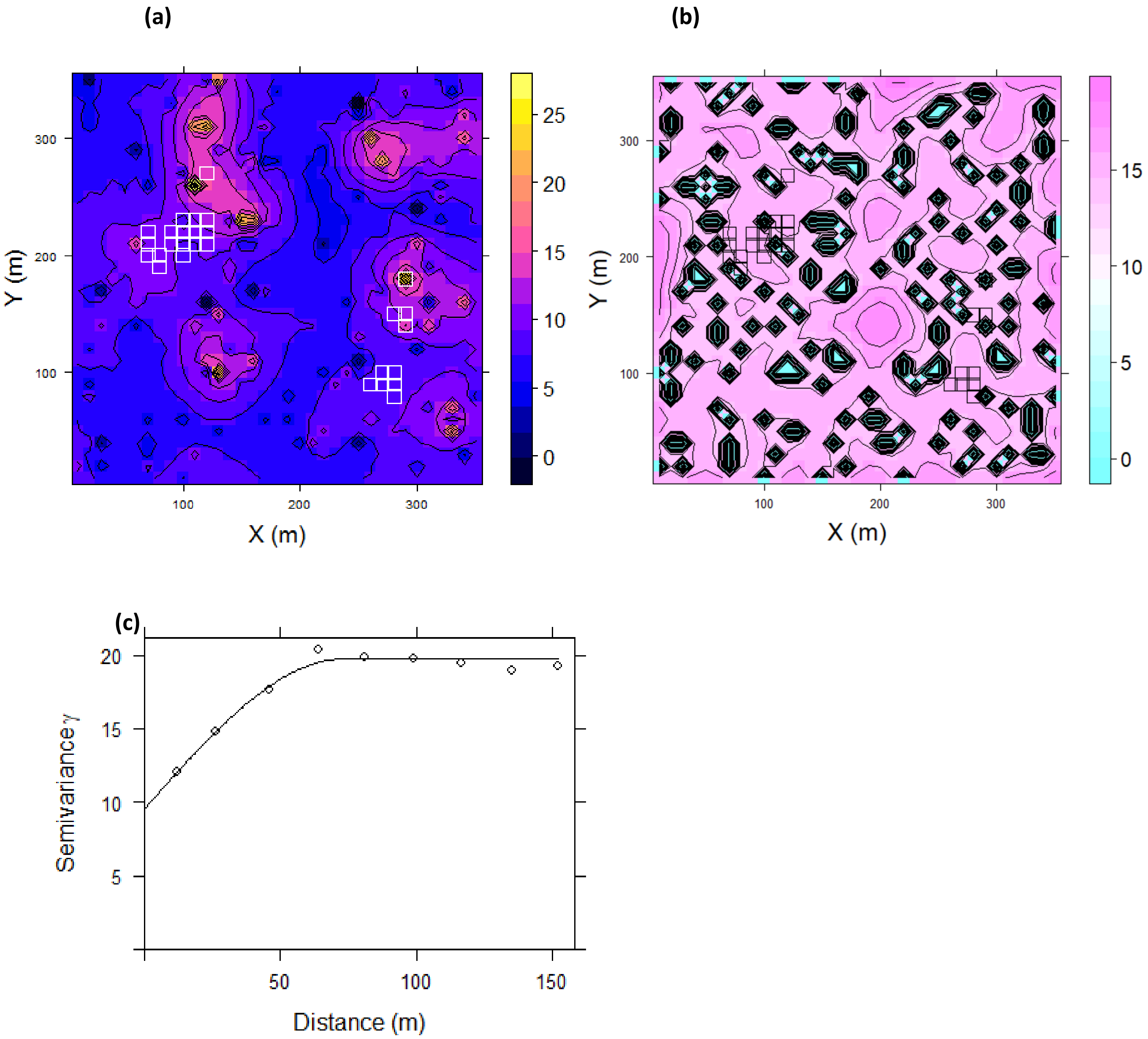

Fig. S8. Kriging heat and contour maps of % organic matter in September 2013 at the sediment's surface (top 0-5 cm) around the oyster reefs (white 10 * 10 m squares that are >50 % occupied by reef). (A) Prediction, (B) prediction variance, (C) model variogram. Deeper (5-10 cm depth) OM and OM from 2014 (besides a negative x-trend) showed little spatial structure so could not be Kriged.

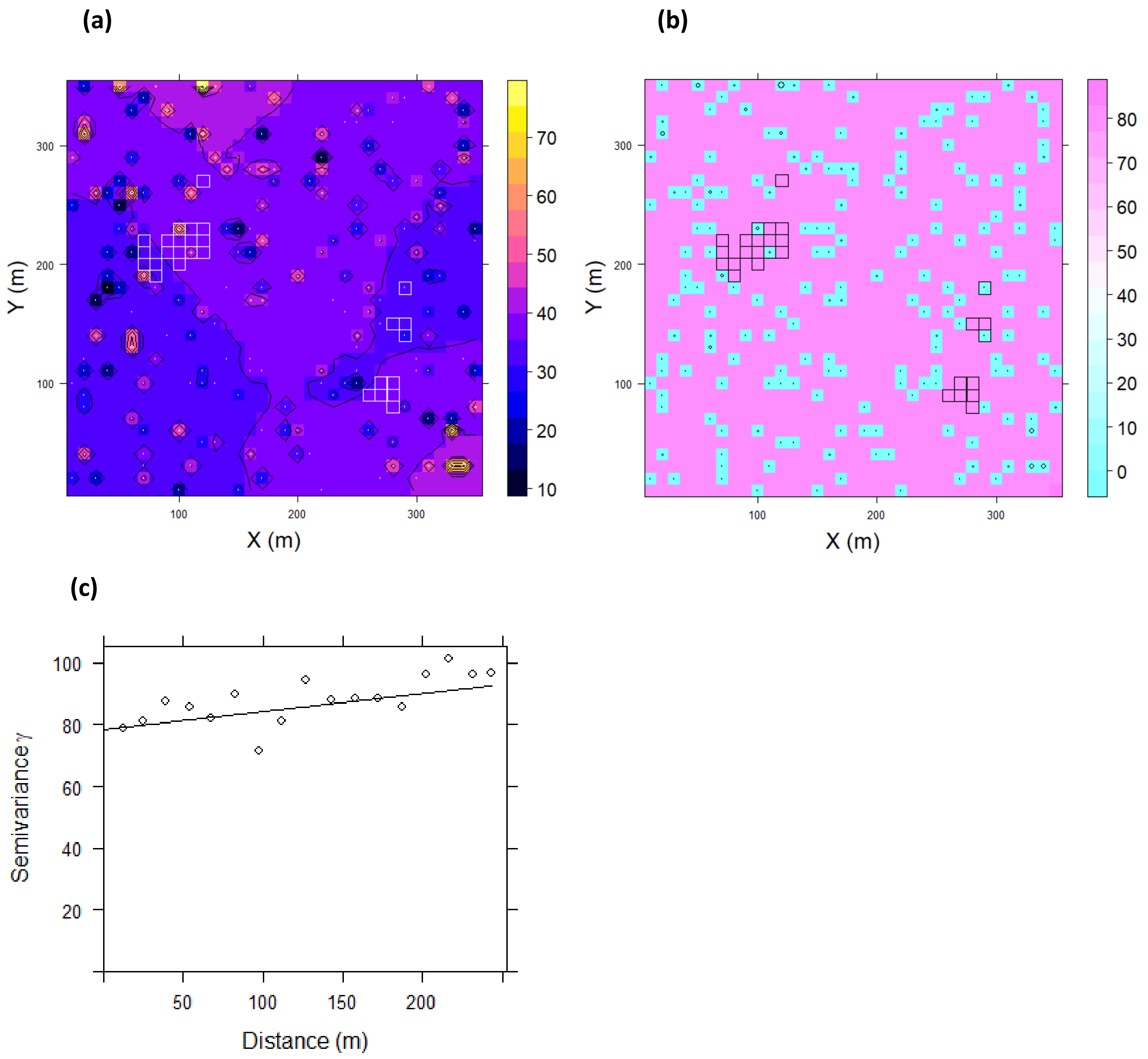

Fig. S9 Kriging heat and contour maps of sediment median grain size in September 2013 at the sediment's surface (top 0-5 cm) around the oyster reefs (white 10 * 10 m squares that are >50 % occupied by reef). (A) Prediction, (B) prediction variance, (C) model variogram.

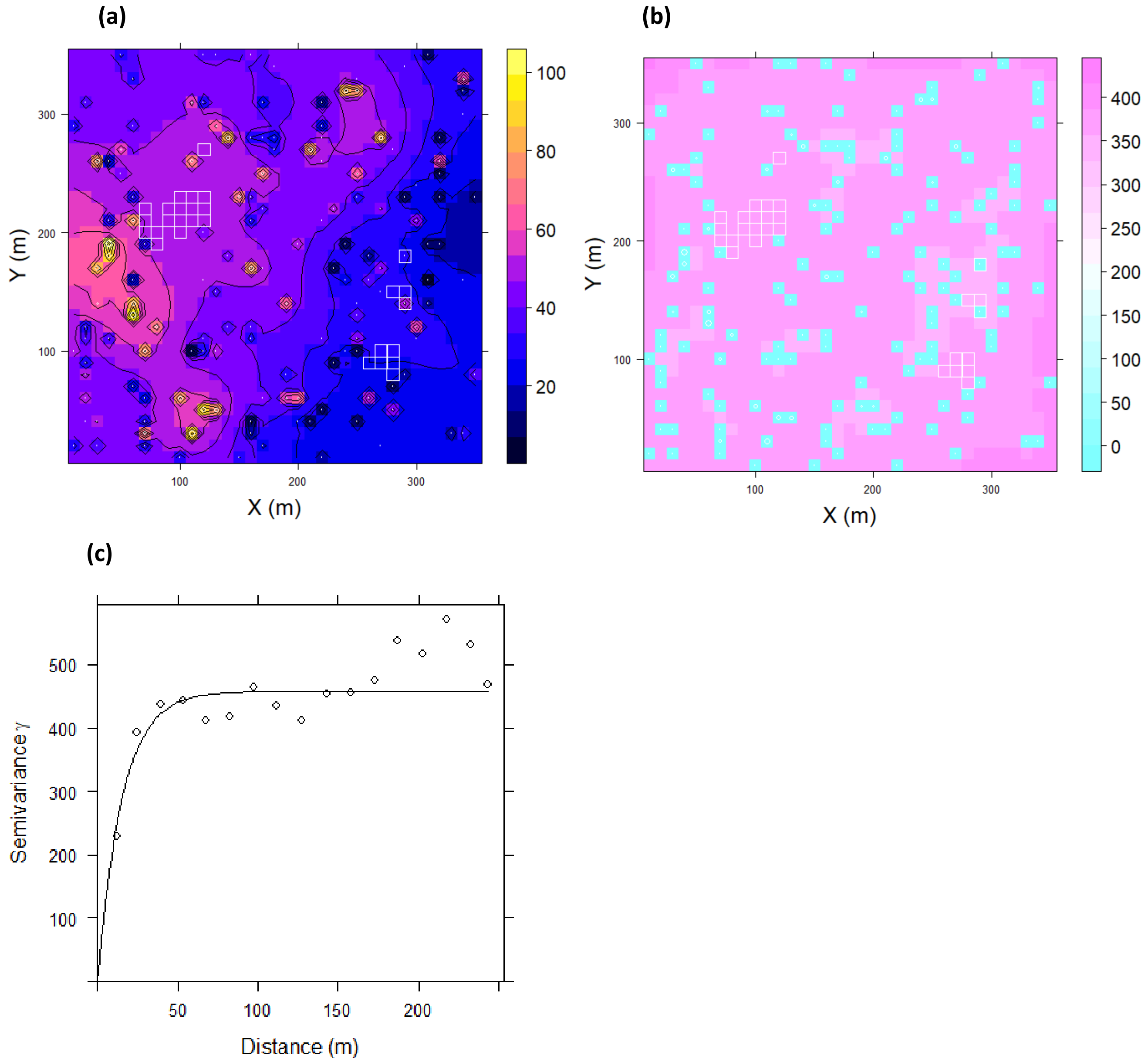

Fig. S10 Kriging heat and contour maps of sediment median grain size in October 2014 at the sediment's surface (top 0-5 cm) around the oyster reefs (white 10 * 10 m squares that are >50 % occupied by reef). (A) Prediction, (B) prediction variance, (C) model variogram.

Macrofauna details

During the summer 2013 sampling campaign alone, 9980 individuals from 27 macrofaunal species were counted, with the most abundant three species, *P. ulvae*, *M. balthica* and *S. plana*, comprising 87.9 % of the total abundance, while 22 species each comprised <1 % of the total abundance (listed in Table S3). Of the total macrofaunal abundance, five species contributed >1 % each: the deposit feeder, *P. ulvae*, 38.3 %; the facultative suspension feeders, *M. balthica*, 37.0 % and *S. plana*, 12.6 %; the predators, *R. obtusa*, 6.2 %, and *N. hombergii*, 4.6 %. Biomass data for these dominant five species was thus sufficient to explore spatial structure, except for the polychaete *N. hombergii*, which despite careful sampling was frequently fragmented, and summed length per sample was instead used. The correlation between *Nephtys* abundance and sum length was high r = 0.879 (similar to that observed between other taxa's abundance and biomass). Because abundance was used to calculate biomass, these were very strongly correlated (*P. ulvae*, r = 0.984; *M. balthica*, r = 0.898; *S. plana*, r = 0.771).

Table S2. Leave-one-out cross validation of Kriging models for species' biomass. Z score is a standardised residual (accounting for the Kriging variance).

	**Mean z-score (ideally 0)**	**Variance z-score (ideally 1)**	**Correlation observed and predicted (ideally 1)**
**Nematodes**	0.001	0.98	0.41
M. balthica	0.001	0.86	0.61
S. plana	0.001	0.86	0.82
P. ulvae	-0.001	1.03	0.68
N. hombergii	0.000	0.98	0.31
Biomass2013	0.002	0.93	0.72
Biomass2014	0.001	0.99	0.69

Individual species spatial distributions

Broad-scale spatial trends were present in biomass patterns, with four of five species being significantly related to the Y axis (bathymetry r_s = 0.06, 0.08, 0.35, 0.72, respectively, for *P. ulvae*, *R. obtusa*, *M. balthica* and *S. plana*, Table 4), and both *P. ulvae* and *R. obtusa* significantly related to the X axis (r_s = 0.29, 0.28; Table 4). Spatial autocorrelation was modelled by semivariograms, with spherical plus nugget models providing satisfactory fits for *M. balthica, S. plana* and *P. ulvae*. *P. ulvae* showed anisotropy, modelled by an ellipse with main axis at 45° relative to the Y axis and an anisotropy ratio of 0.6.

Despite different diets, the MPB grazer *P. ulvae* and the predator *N. hombergii*, both showed clear spatial interactions (low biomass) with the reefs and MPB patterns (Figs. S12 & S13, Table 4). The Kriging model for *Nephtys* had high variance and cross validation suggested the model had a poor predictive capacity (Table S2). *Macoma* and *Scrobicularia*, both facultative suspension feeders, showed different severity of trends with Y axis (bathymetry), which affected the clarity of their relations to MPB and distance from the reefs (Figs. S14 & S15, Table 4). *R. obtusa* biomass was uncorrelated with MPB or distance from oyster reefs or the rock, but was weakly correlated with biomass patterns of its prey, *P. ulvae* (Table 4). However, once trends were accounted for, little spatial structure remained in *R. obtusa* biomass, so Kriging was not supported.

Figure S11. [On next page] Kriging variance and variograms for Figure 4, for 2013 biomass (a and b), 2014 biomass (c and d), 2013 Simpson's diversity (e and f). Kriging prediction plots and unit information in Fig. 4.

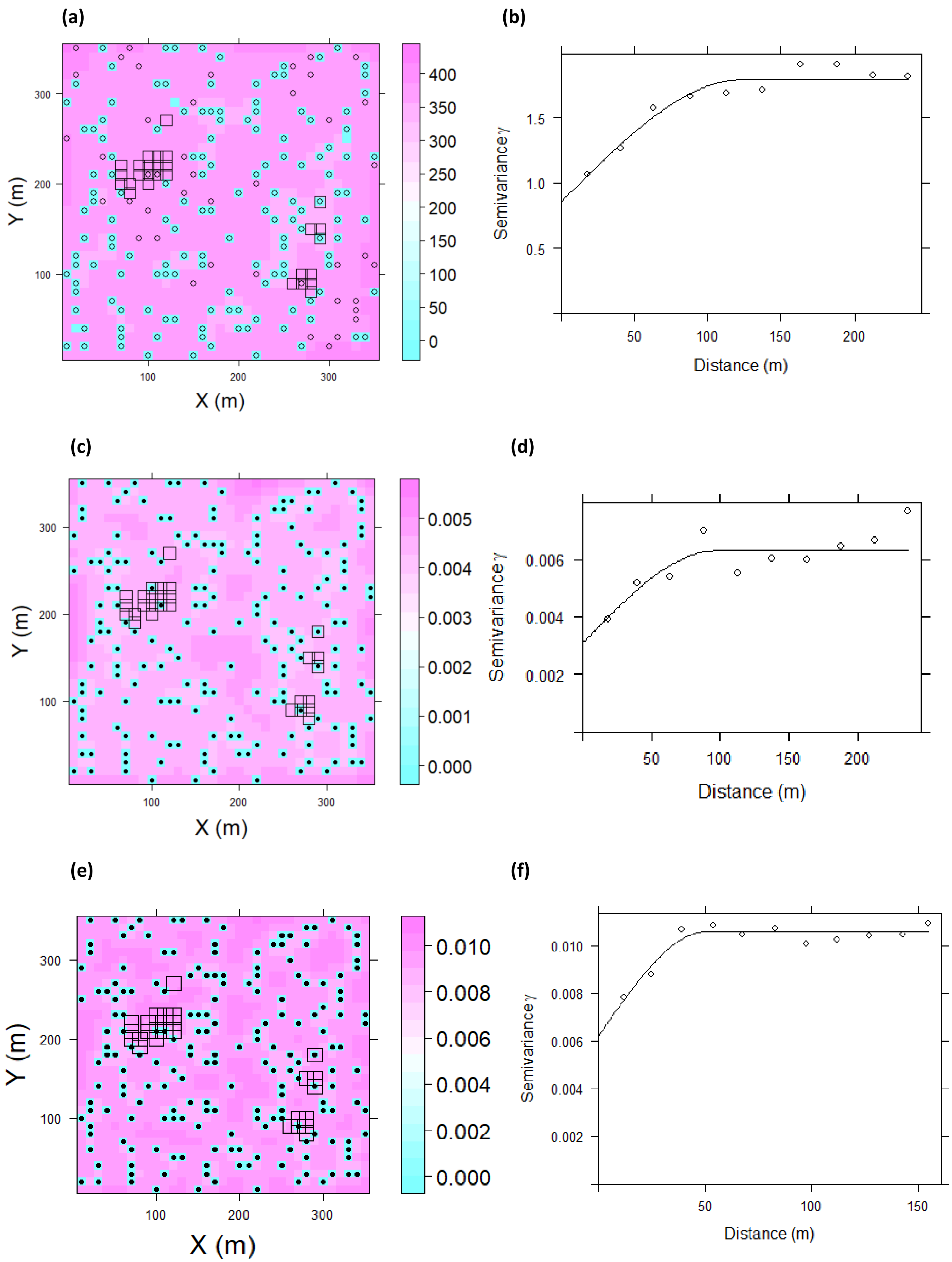
(a)
(b)
Y (m)
X (m)
100
200
300
400
350
300
250
200
150
100
50
0
Semivariance γ
Distance (m)
1.5
1.0
0.5
50
100
150
200
(c)
(d)
0.005
0.004
0.003
0.002
0.001
0.000
0.006
0.004
0.002
(e)
(f)
0.010
0.008
0.006
0.004
0.002
0.000

Table S3. Pre-treatment taxon total abundances across samples from summer 2013.

Macrofaunal species	Occurrence
Peringia ulvae	3820
Macoma balthica	3693
Scrobicularia plana	1256
Retusa obtusa	623
Nephtys hombergii	460
Crangon crangon	19
Diopatra neapolitana	16
Cerastoderma sp.	15
Corophium volutator	14
Venerupis philippirum	11
Polydora sp.	8
Turbonilla acuta	7
Heteromastus filiformis	6
Kurtiella bidentata	5
Veneridae	5
Gammarus locusta	3
Abra nitida	3
Venerupis sp.	3
Cirratulidae	2
Palaemonidae	2
Lagis koreni	2
Diptera larva	2
Hediste diversicolor	2
Copepoda	1
Cerastoderma glaucum	1
Monocorophium acherusicum	1
Carcinus maenas	1

Meiofaunal taxon	Occurrence
Nematoda	77088
Copepoda	3624
(Nauplius)	570
Polychaeta	215
Cnidaria	102
Oligochaeta	63
Halacarida	11
Kinorhyncha	8

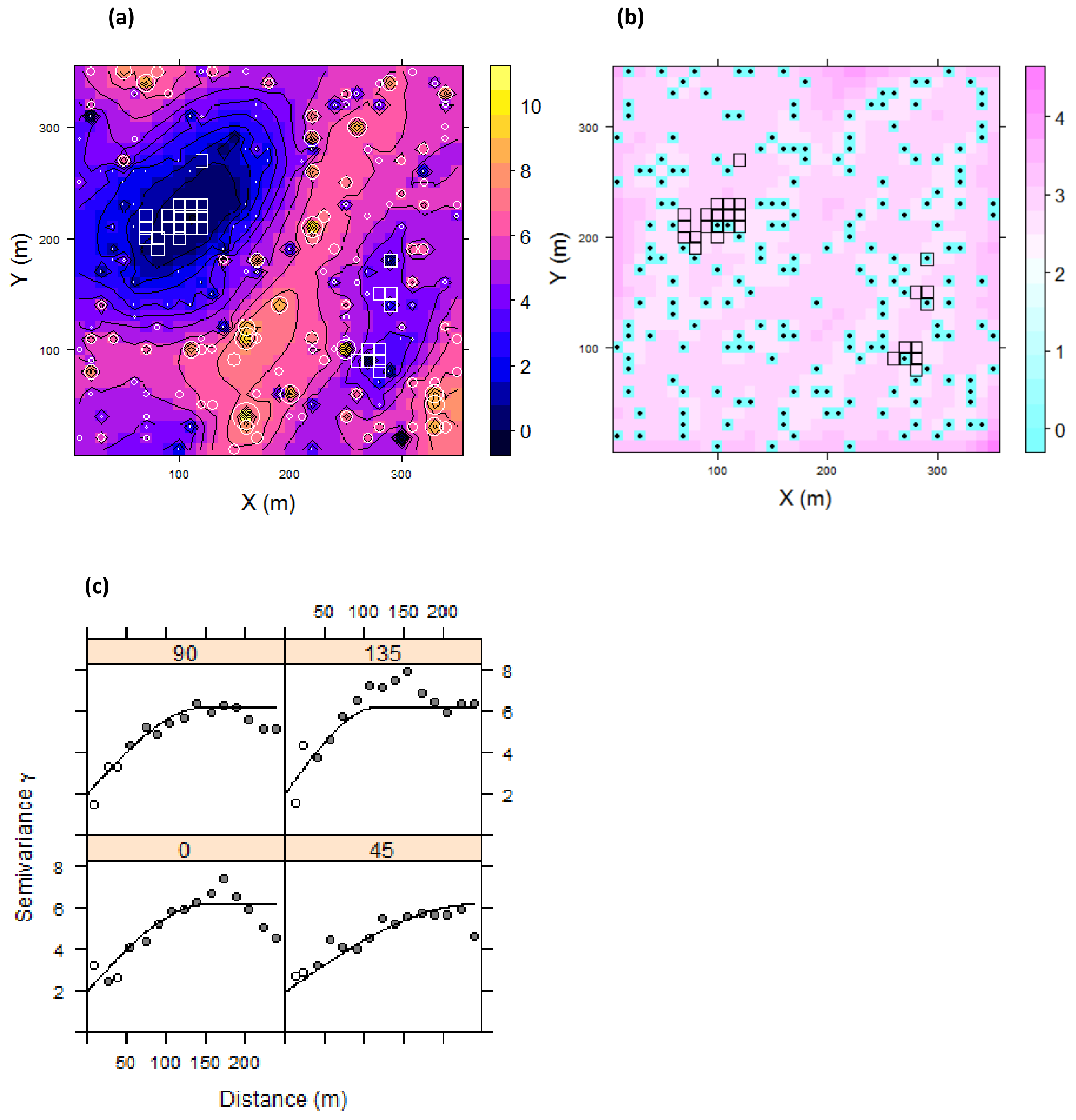

Figure S12. Kriging heat and contour map of *Peringia ulvae* (formerly *Hydrobia*) biomass per cell around the oyster reefs (white squares). (A) Prediction, (B) prediction variance, (C) model variogram showing how the elliptical range accounts for anisotropy. Data and scale bar are square root transformed. Significant trends with both X and Y axis. Other details as in Fig. S8.

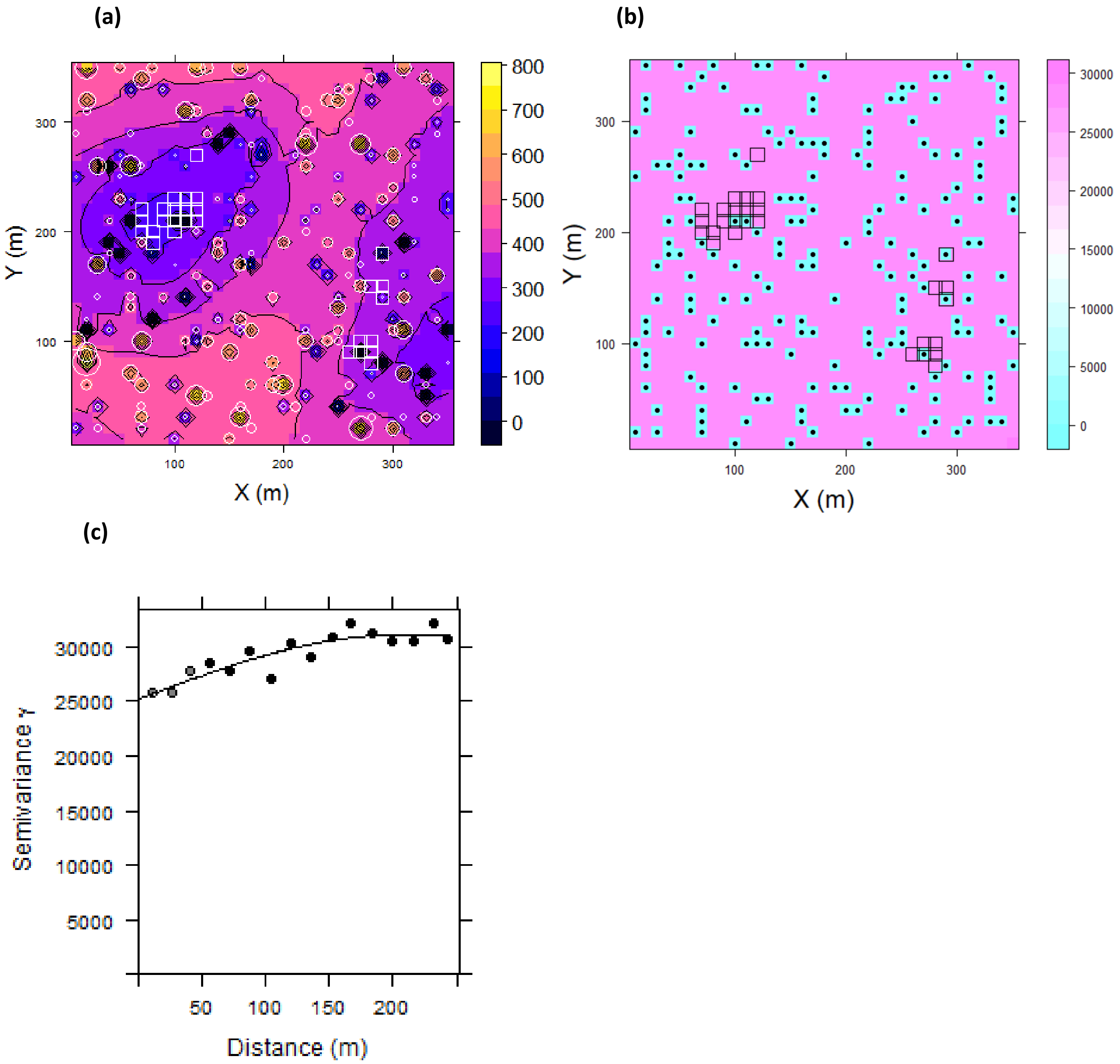

Figure S13. Kriging heat and contour map of *Nephtys hombergii* cumulative length per cell around the oyster reefs (white squares). (A) Prediction, (B) prediction variance, (C) model variogram. Data and scale bar are square root transformed. Other details as in Fig. S8.

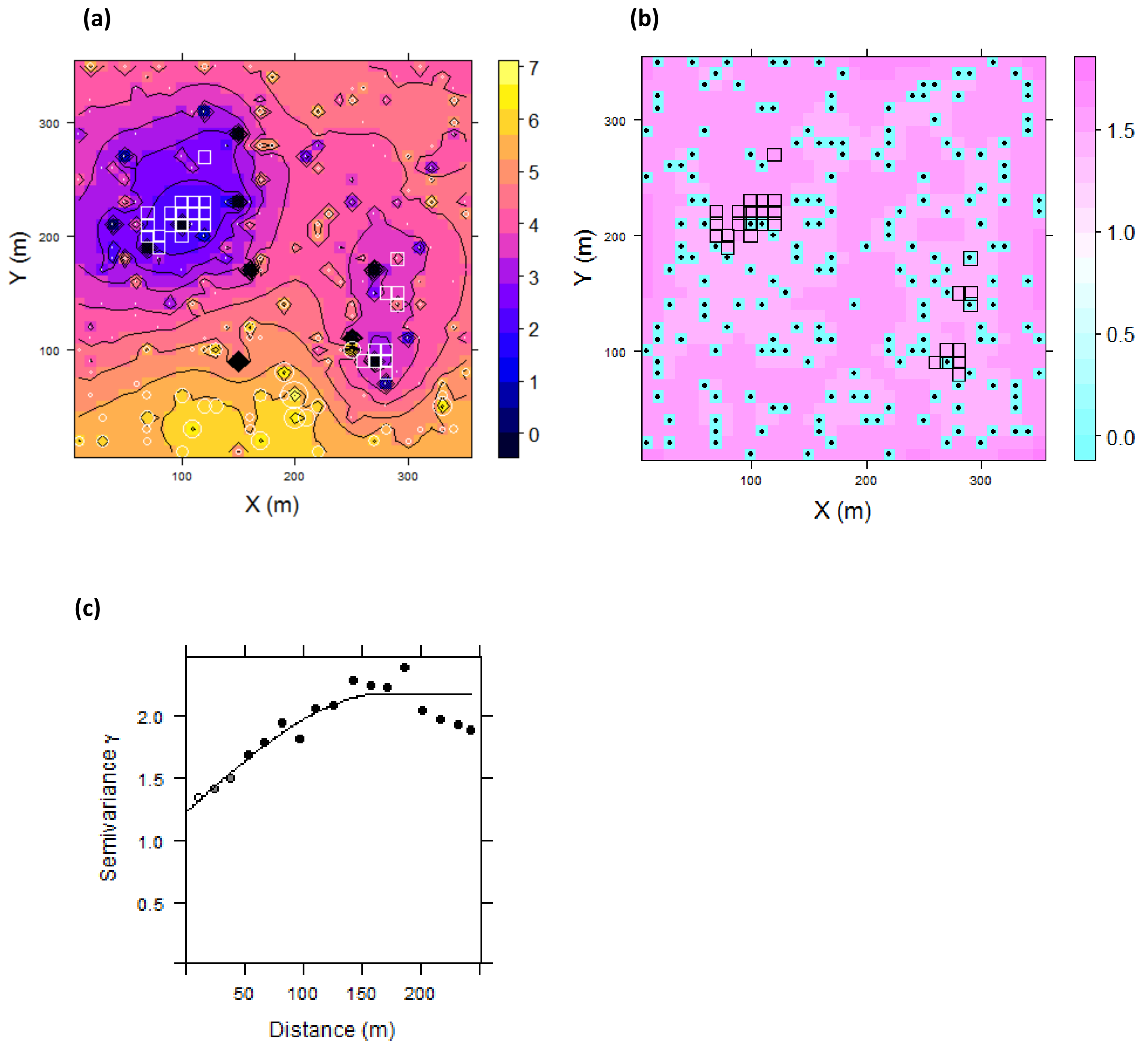

Figure S14. Kriging heat and contour maps of *Macoma balthica* biomass per cell around the oyster reefs (white squares). (A) Prediction, (B) prediction variance, (C) model variogram. Trends with Y axis (bathymetry). Data and scale bar are LN transformed. Other details as in Fig. S8.

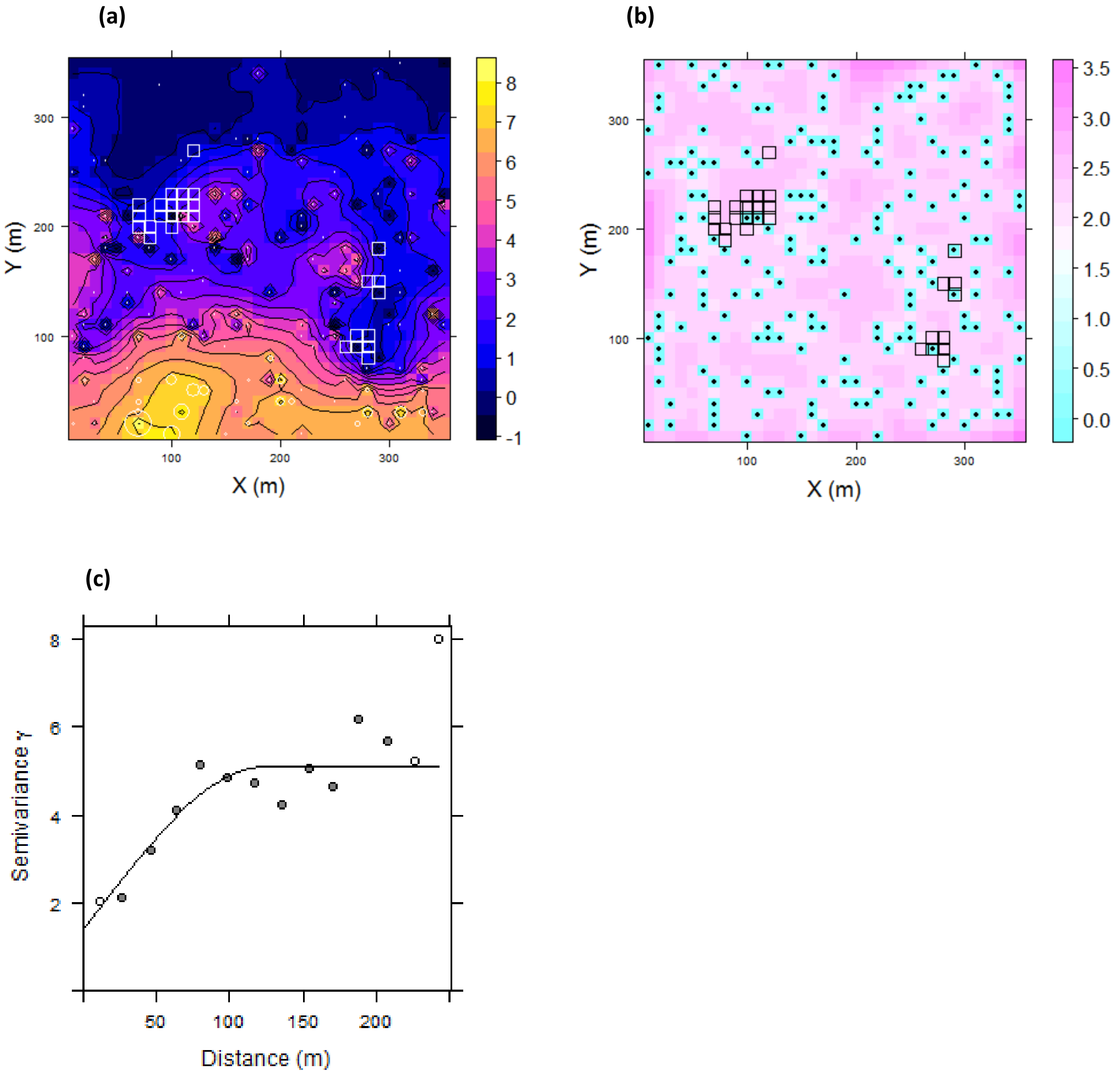

Figure S15. Kriging heat and contour maps of *Scrobicularia plana* biomass per cell around the oyster reefs (white squares). (A) Prediction, (B) prediction variance, (C) model variogram. Strong trend with Y axis (bathymetry). Data and scale bar are LN transformed. Other details as in Fig. S8.

Comparing pre-treatment biomasses

Sampled infauna biomasses were extrapolated from sample means per metre to the area of the overall sampling grid. Biomass for the *C. gigas* oyster reef was estimated by remote sensing (4) and converted from wet weight to ash-free dry weight (AFDW; 5) to be comparable with other species.

Biomasses of facultative suspension feeders *Scrobicularia* (despite its steep bathymetric trend) and *Macoma* were of the same order of magnitude as the oysters of the reefs (Table S4). *P. ulvae* biomass was an order lower than *S. plana* and *M. balthica.* Obligate suspension feeding species (*Cerastoderma edulis* and Venerid clams), potential competitors of the oysters, were very low (Table S4).

Table S4. Comparing pre-treatment biomasses of the dominant fauna in the sampling grid.

Species	Estimated mass (kg AFDW)
Crassostrea gigas	255-340
Scrobicularia plana	200.0
Macoma balthica	152.9
Peringia ulvae	33.5
Veneridae	3.9
Cerastoderma sp.	2.3
Retusa obtusa	0.9

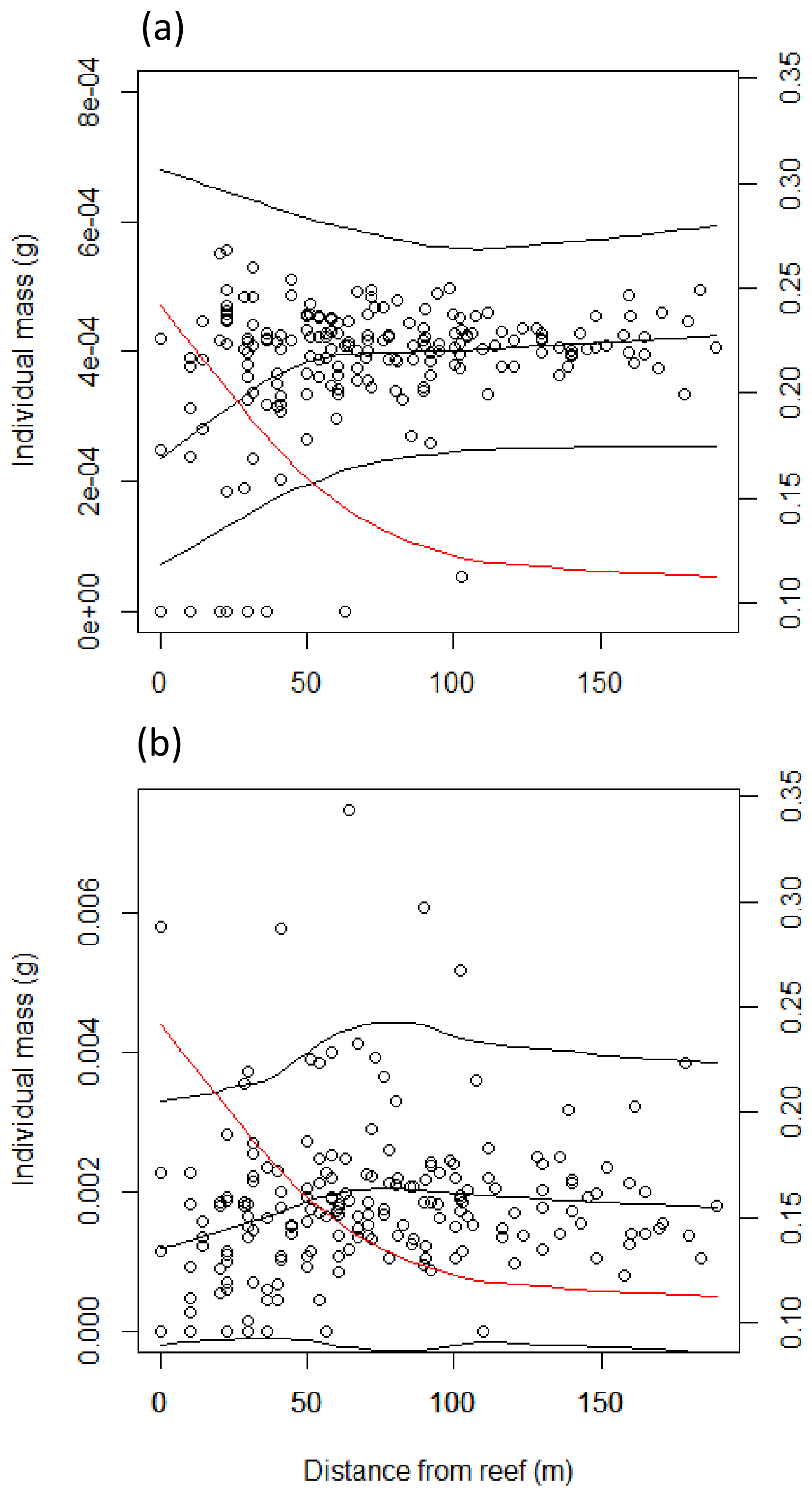

Fig. S16. The spatial footprint of *C. gigas* reefs on the individual size of mudflat grazers, (a) *P. ulvae* and (b) *M. balthica*, and MPB in the vicinity of 10s of m. Black lines are upper and lower 95% central interval around the mean, while the red line is MPB via NDVI with values on the right-hand y axis.

Reef epifauna details

In 2017, the control reef was covered 51.5 ± 39.2 % by *C. gigas,* the residual surface comprising either mud or rock. Reefs were characterised by a species-poor rocky shore assemblage (e.g. *Patella vulgata, Littorina littorea*), with exposed rock and oyster shell being dominated by barnacles (78.3 ± 21.6 %; Table S5).

Net catches were dominated by *Crangon crangon* and *Carcinus maenas* and also included fish: five *Pomatoschistus minutus* with total lengths ranging between 31 – 54 mm; three *Solea solea* with total lengths ranging between 81 – 91 mm; four *Palaemon* sp. carapace with lengths 6 – 7 mm, and one *Hemigrapsus takanoi* (carapace width 9 mm).

Table S5. Quadrat survey results of sessile (%) and mobile (n. individuals) epifauna of the oyster control reef from 2017. CW = carapace width

Species	Unit	Mean per quadrat	SD
S. balanoides	%	78.25	21.60
C. gigas	%	51.50	39.21
Halichondria panicea	%	8.50	24.77
Actinia equina	%	0.55	1.54
L. littorea	Individuals	11.60	9.11
P. vulgata	Individuals	6.65	9.00
Actinia equina	Individuals	2.50	3.85
M. edulis	Individuals	1.15	2.58
N. lapillus	Individuals	0.25	0.72
C. maenas	Individuals	0.20	0.41
Palaemon	Individuals	0.15	0.67
G. umilicalis	Individuals	0.10	0.31
Hemigrapsus	Individuals	0.10	0.31
Venerupsis sp.	Individuals	0.10	0.31
Hemigrapsus	CW mm	17.00	11.31
C. maenas	CW mm	13.75	7.50

Meiofauna details

>80,000 meiofaunal individuals from eight broad taxa were counted over half the sample points (98) of macrofauna. 99 % of individuals were nematodes and copepods, the spatial patterns of which were moderately correlated (Table 4). Nematode abundance showed two abundance troughs and, also for copepod abundance, did not have a clear relationship to the oyster reefs nor to MPB patterns (Fig. S17, Table 4). The nematode Kriging model showed high variance and leave-one-out cross validation suggested that the Kriging model had relatively poor predictive performance. Copepods showed little spatial structure, which precluded variogram mapping and Kriging.

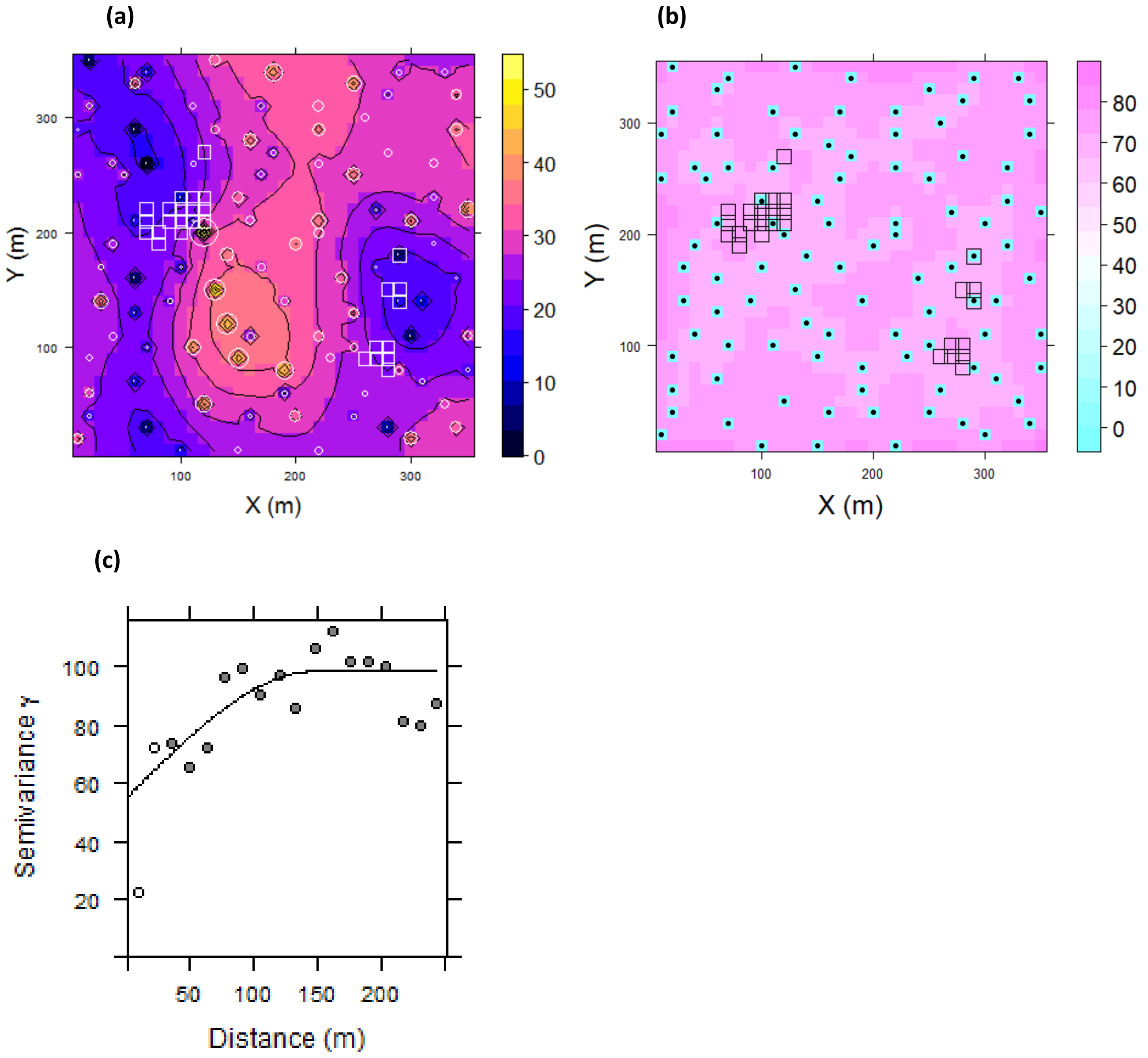

Fig. S17. Kriging heat and contour map of nematode abundance per sample in September 2013 around the oyster reefs (white squares). (A) Prediction, (B) prediction variance, (C) model variogram. Data and scale bar are square root transformed. Copepoda *(not shown)* abundances showed little spatial structure but were moderately correlated with nematode abundance, $r = 0.52$, $p < 0.001$ (df = 76.8). Other details as in Fig. S8.

The timescale of the impact

The burning of the reef was expected to have immediate 'pulse' impacts from the high temperatures reached and extreme conditions (nutrients, anoxia) imposed on the immediately surrounding mudflats. This affected organisms with different turnover rates, resulting in 'impacts' at different temporal scales. For instance, the turnover of MPB is expected to be a matter of days in summer and thus having recovered from the burning by the sampling dates (see Fig. S7). Occupant mobile organisms, such as crabs and gastropods (e.g. *P. ulvae*) are likely to have been initially killed but their numbers may have partially or completely recovered due to immigration, although the rate and extent of recovery is unclear. *P. ulvae* abundances appear unchanged in 2014, suggesting recovery, but those of *M. balthica* remain significantly lower (Table 2). In 2014, the predation halo (under hypothesis 3) around the treatment reef is less clear than in 2013 (Fig. 4b and c), which may suggest that crab predation had not yet recovered by the autumn 2014 sampling. Long-lived, largely sessile organisms, including the oysters and *S. plana* are likely to take one or more years to recover, especially the recovery of large individual numbers. This experiment therefore has the opportunity to compare the impacts of the loss of the oysters, other sessile reef-dwelling fauna, and immediately-situated infaunal bivalves on the MPB biomass. The killing of the oysters (and other reef organisms) was unlikely to be an isolated effect of burning: instead, the addition of large quantities of inorganic C and nutrients from the burnt remains of organisms and the straw is likely, which would be widely distributed by tidal currents. The duration of this enrichment effect is likely to be weeks to months, and an initial MPB growth pulse across the grid was confirmed by Echappé et al. (3). Over time, waves and bioturbation are likely to redistribute and bury burning residue, shutting it away from all but bacteria, their consumers and deep deposit feeders, although the timeframe is unclear. Wind speed was not especially marked during the 81 days from impact to sampling but this duration is expected to result in considerable horizontal and vertical movement of the burning residue.

Appendix S2: Supplementary Methods

Wider situation of the reefs

We describe patches of high MPB production in the summer in Bourgneuf Bay, which largely collapsed in winter. Guarini et al. (6) found their broad-scale patches to expand in June (increasing variogram range) and to shrink by January, while maximum levels remained consistent throughout the year. Peak MPB biomass in our finer scaled study, however, was not seasonally consistent, and decreased substantially in winter, likely because of the small size of our oyster reefs being more susceptible to water currents. Larger oyster reefs nearby appeared to maintain high winter MPB biomass (3).

Details of the reef burning

Although we did not sample structural complexity of the reefs before vs after the burning, the difference between dead gaping oysters and oysters gaping to feed (alongside the pumping of water) on flood tide currents is unclear. At the broad spatial resolution here of 10s m, this difference is not expected to be important, though at finer scales, water movement differences may arise.

The many different variables sampled in this study allow us to investigate many possibilities of experimental artefact (see also 3). For example, while shallow sediment organic matter rose from 2013 into 2014, the only spatial structure detected was a negative x-trend (from mean 13% to 10.1%), with highest % organic matter furthest from the treatment reef and no noticeable concentration around it.

A. 16th July 2014

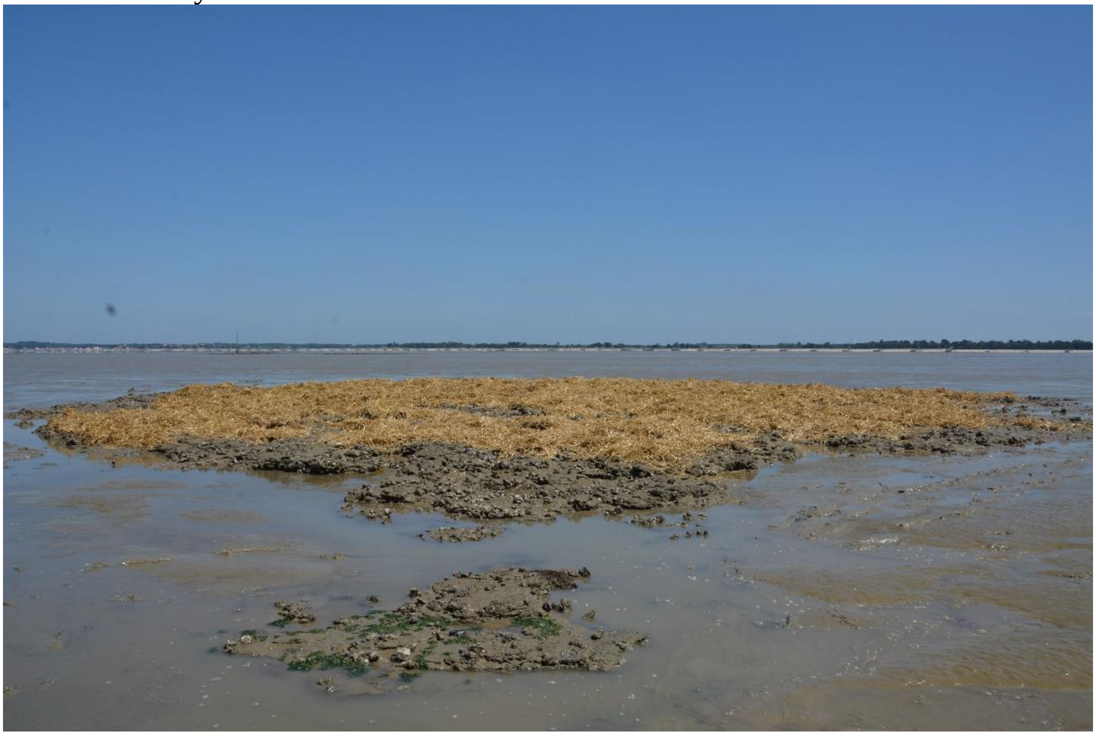

B. 16th July 2014

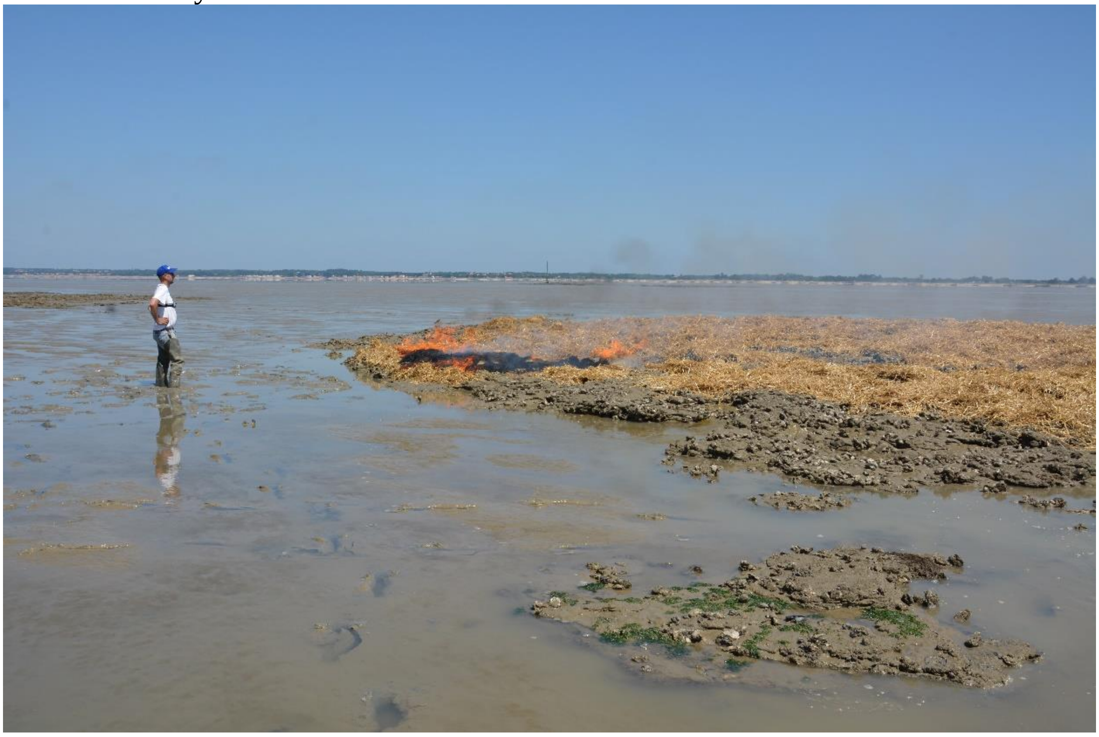

C. 17th July 2014

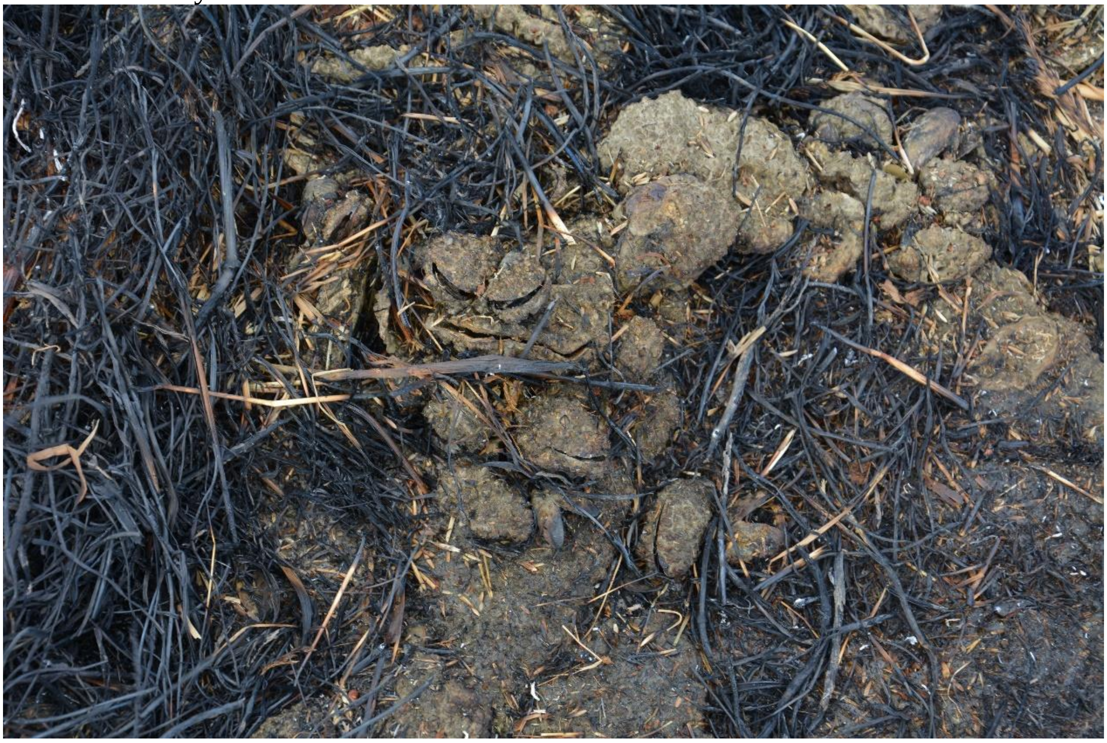

D. 17th July 2014

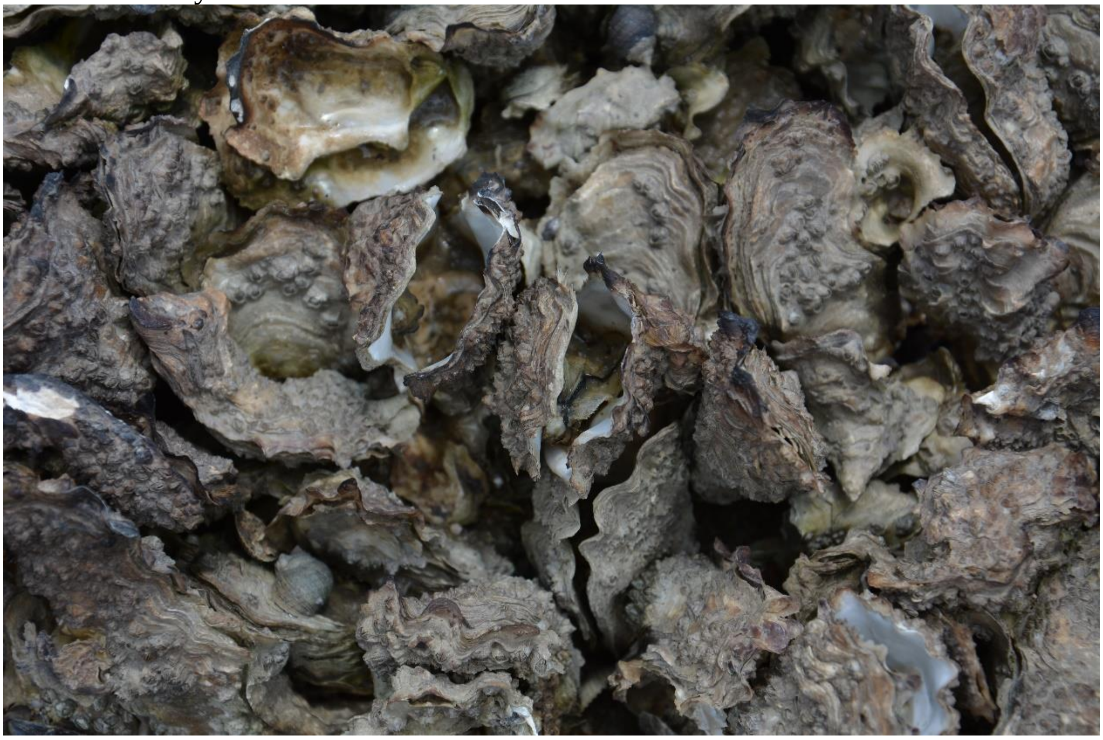

E. 8th October 2014

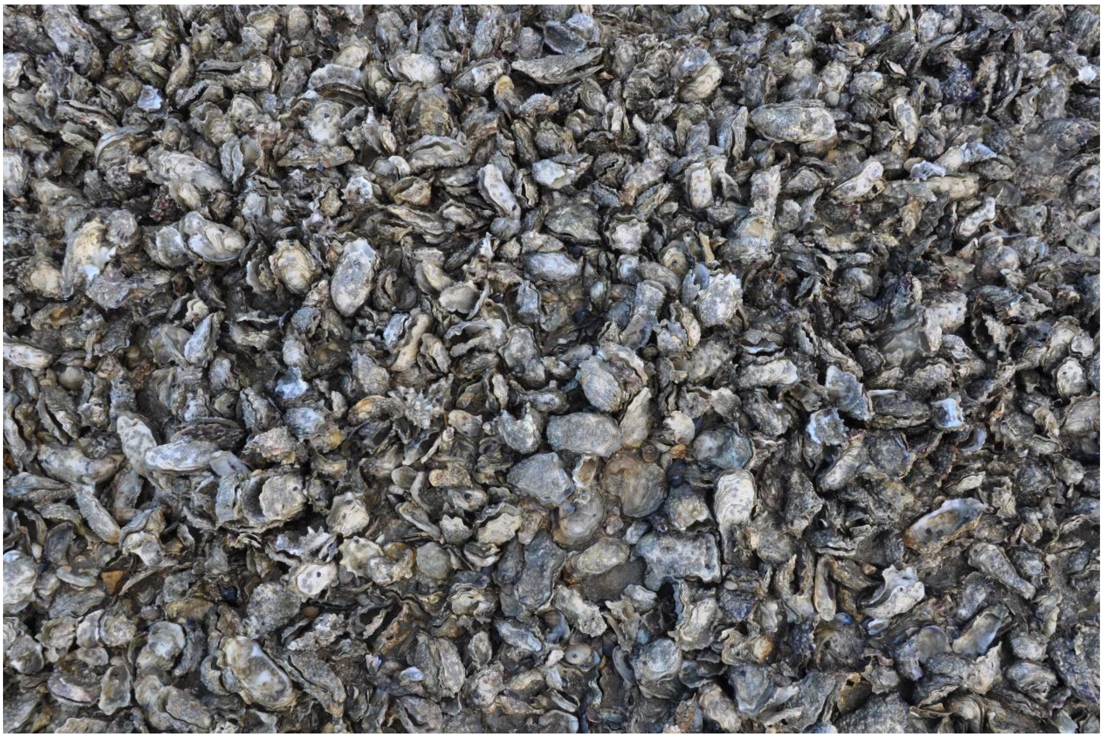

Figure S18. Photos taken of the burning of the treatment reef (A, B) and its impacts on the oysters immediately (C, D) and by the time of post-treatment sampling (E).

SEM

The *oyster biodeposition* hypothesis (1) expected high MPB in the immediate surroundings of the reef alongside an increase in OM and accordingly a decrease in MGS. Evidence for the *abiotic* hypothesis (2) was anticipated as decreasing sediment MGS and increasing MPB with proximity to the reef, which would be unaffected by the manipulation. For the *predation* hypothesis (3), MPB is expected to respond only to changes in the grazers, and be released from grazing pressure nearer the reefs because of increased crab foraging. This suppresses the biomass and body sizes of epifaunal gastropods and infaunal bivalves, which graze MPB. The hypothesis (3) treatment response also depended on whether the infauna were affected by the burning and, if they were not, whether predators had successfully recolonised the reef by the sampling date. Hypothesis (4), *meiofauna*, assumed a direct link between abundance of nematodes and MPB, potentially mediated by copepod distribution and MGS, regardless of the treatment impact, so was tested in the pre-treatment data from 2013. The R notation of the SEM equations for Fig. 3 A-D was the following:

```
model2013<-'
NDVI ~  ReefDistsBoth + OM + Graz
OM ~ MGS
Graz ~ ReefDistsBoth + MGS
Graz =~ Mac + Scr
Mac ~~ Scr
Mac ~~ Bath
Scr ~~ Bath
OM ~~ OM
NDVI ~~ NDVI
Graz ~~ Graz
'

model2014control<-'
NDVI ~ ReefDistsCon + Graz + OM + MGS
MGS ~ Bath
Graz ~ ReefDistsCon + OM
Graz =~ Mac + Scr
'

model2014both<-'
NDVI ~ ReefDistsBoth + Graz + MGS + OM
MGS ~ Bath
Graz ~ ReefDistsBoth + OM
Graz =~ Mac + Scr
'

modelmeio<-'
NDVI ~ Nematode + OM + ReefDistsBoth
Nematode ~ Copepod + MGS + OM+ ReefDistsBoth
Copepod ~ MGS + OM + ReefDistsBoth
'
```

Geostatistical details

S. plana biomass had near zero values at all cells except in one region of the sampling grid that had very high biomasses. Because this pattern resulted in a highly non-normal data distribution, the variogram was modelled from a spatial subset of the spatial points, excluding most near zero values from the lower bathymetric levels (7). This subset included points within a rectangle 120 * 350 m at the higher bathymetry end of the sampling grid.

Empirical semivariogram clouds and plots were inspected for optimal cut-off, lag and severity of anisotropy. For all semivariograms, a cutoff point of half the diagonal maximum distance (~248m) across the grid was used, while bin widths of 12 m for *P. ulvae* and *M. balthica*, 14 m for *N.*

hombergii, 18 m for *S. plana* and 20 for *R. obtusa* were adopted. To assess cases of anisotropy, empirical semivariograms were checked in the 0, 45, 90 and 135 degree directions relative to the y axis (7). Only *P. ulvae* biomass showed substantial anisotropy, which was modelled using an elliptical rather than a spherical range, with a principle axis of 45° and a severity of 0.6.

Wider acknowledgements

The courageous volunteers included the following: Alexandridis, Nikolaos; Androuin, Thibault; Bacher, Cédric; Benyoucef, Ismaïl; Blanchet, Aline; Buchet, Rémi; Caisey, Xavier; Canvaroue, Ronan; Carlier, Antoine; Castilla-Gavillan, Marta; Cordier, Céline; Cosson, Richard; Cugier, Philippe; Déléris, Paul; Descarrega, Fanny; Drouet, Sigrid; Dumay, Justine; Godet, Laurent; Haberkorn, Hansy; Jauffrais, Thierry; Larnicol, Morgane; Lascours, Carol; Le Bris, Anthony; Lépinay, Alexandra; Lerouxel, Astrid; Martin-Jézéquel, Véronique; Morançais, Michèle; Moreau, Cristof; Navarro, Angelica; Pardo, Sophie; Robert, Alex; Thomas, Yoann; Volkenborn, Nils.

Supplementary references

1. V. Méléder, *et al.*, Spatio-temporal changes in microphytobenthos structure analysed by pigment composition in a macrotidal flat (Bourgneuf Bay, France). *Mar. Ecol. Prog. Ser.* **297**, 83–99 (2005).

2. P. Cartaxana, B. Jesus, V. Brotas, Pheophorbide and pheophytin a-like pigments as useful markers for intertidal microphytobenthos grazing by Hydrobia ulvae. *Estuar. Coast. Shelf Sci.* **58**, 293–297 (2003).

3. C. Echappé, *et al.*, Satellite remote sensing reveals a positive impact of living oyster reefs on microalgal biofilm development. *Biogeosciences* **15**, 905–918 (2018).

4. A. Le Bris, *et al.*, Hyperspectral remote sensing of wild oyster reefs. *Estuar. Coast. Shelf Sci.* **172**, 1–12 (2016).

5. A. Ricciardi, E. Bourget, Weight-to-weight conversion factors for marine benthic macroinvertebrates. **163**, 245–251 (1998).

6. J. M. Guarini, *et al.*, Dynamics of spatial patterns of microphytobenthic biomass: inferences from a geostatistical analyisis of two comprenhensive surveys in Marennes-Oléron Bay (France). *Mar. Ecol. Prog. Ser.* **166**, 131–141 (1998).

7. R. S. Bivand, E. Pebesma, V. Gomez-Rubio, *Applied Spatial Data Analysis in R* (Springer Science & Business Media, 2013).
