# Supplementary material for: Extensive spatial impacts of oyster reefs on an intertidal mudflat community via predator facilitation
Source: Commun Biol. 2022 Mar 22;5:250. doi: 10.1038/s42003-022-03192-4 (PMC8940938; doi:10.1038/s42003-022-03192-4)
Supplement: Supplementary file 3 — Reporting Summary [file 42003_2022_3192_MOESM3_ESM.pdf]

nature research

Corresponding author(s): Carl J Reddin

Last updated by author(s): Jan 25, 2022

Reporting Summary

Nature Research wishes to improve the reproducibility of the work that we publish. This form provides structure for consistency and transparency in reporting. For further information on Nature Research policies, see our Editorial Policies and the Editorial Policy Checklist.

Statistics

For all statistical analyses, confirm that the following items are present in the figure legend, table legend, main text, or Methods section.

n/a	Confirmed	
☐	☒	The exact sample size (n) for each experimental group/condition, given as a discrete number and unit of measurement
☐	☒	A statement on whether measurements were taken from distinct samples or whether the same sample was measured repeatedly
☐	☒	The statistical test(s) used AND whether they are one- or two-sided<br>*Only common tests should be described solely by name; describe more complex techniques in the Methods section.*
☐	☒	A description of all covariates tested
☐	☒	A description of any assumptions or corrections, such as tests of normality and adjustment for multiple comparisons
☐	☒	A full description of the statistical parameters including central tendency (e.g. means) or other basic estimates (e.g. regression coefficient) AND variation (e.g. standard deviation) or associated estimates of uncertainty (e.g. confidence intervals)
☐	☒	For null hypothesis testing, the test statistic (e.g. F, t, r) with confidence intervals, effect sizes, degrees of freedom and P value noted<br>*Give P values as exact values whenever suitable.*
☒	☐	For Bayesian analysis, information on the choice of priors and Markov chain Monte Carlo settings
☐	☒	For hierarchical and complex designs, identification of the appropriate level for tests and full reporting of outcomes
☐	☒	Estimates of effect sizes (e.g. Cohen's d, Pearson's r), indicating how they were calculated

Our web collection on statistics for biologists contains articles on many of the points above.

Software and code

Policy information about availability of computer code

Data collection	No software was used
Data analysis	All mapping and analyses were performed in the statistical computing environment R, with some analyses using the referenced packages lavaan (v0.6-7) and gstat (v2.0-6).

For manuscripts utilizing custom algorithms or software that are central to the research but not yet described in published literature, software must be made available to editors and reviewers. We strongly encourage code deposition in a community repository (e.g. GitHub). See the Nature Research guidelines for submitting code & software for further information.

Data

Policy information about availability of data

All manuscripts must include a data availability statement. This statement should provide the following information, where applicable:

- Accession codes, unique identifiers, or web links for publicly available datasets
- A list of figures that have associated raw data
- A description of any restrictions on data availability

The datasets analysed during the current study are available in the Zenodo repository, doi: 10.5281/zenodo.5902388. This includes data supporting figures 1-5. All used software was publicly available and described in the Methods. No other custom code was used besides the function 'lavSpatialCorrect' by Jarrett Byrnes (https://github.com/jebyrnes/spatial_correction_lavaan).

Field-specific reporting

Please select the one below that is the best fit for your research. If you are not sure, read the appropriate sections before making your selection.

☐ Life sciences ☐ Behavioural & social sciences ☒ Ecological, evolutionary & environmental sciences

For a reference copy of the document with all sections, see nature.com/documents/nr-reporting-summary-flat.pdf

Ecological, evolutionary & environmental sciences study design

All studies must disclose on these points even when the disclosure is negative.

Study description	We sampled mudflats of Bourgneuf Bay, northwest France, 360° in the near (10s of m) and far (100s of m) surroundings of two oyster reefs, each > 750 m2, over two seasons for sediment, MPB, meiofauna and macrofauna on a regular spatial grid, 196 sample stations for most variables (half this number for meiofauna). We sampled before and after a large before-after-control-impact (BACI) experiment, sacrificing the oysters of one reef while maintaining the other reef as a control.
Research sample	Several variables were sampled on the mudflats surrounding these oyster reefs, including MPB NDVI, MPB pigment composition and biomass, mudflat macrofauna and meiofauna, granulometry (median grain size), sediment organic matter (%), geographical and bathymetric coordinates, oyster reef biomass, reef epifauna, Fyke nets for mobile fauna.
Sampling strategy	196 sampling stations were randomly determined within cells of a regular spatial grid surrounding the oyster reefs, which were sampled at dates over two early autumn (one before, one after the BACI) and one winter season. Satellite data was limited by satellite orbit frequency, quality criteria, and image resolution. Several additional aspects were sampled on different schemes or subsets of these sampling stations, all fully detailed in the Methods section.
Data collection	Cores were taken on the mudflats by all co-authors. A summary of study variables and their sampling methodologies is available in Table 3.
Timing and spatial scale	The 350 x 350 m grid was sampled for all variables during the winter MPB low and early autumn peak seasons, on the dates 18-19th September 2013 and 17-18th March 2014, before treatment, and on 7-8th October 2014 after treatment, generally with a spatial lag of 10 m between samples. To estimate the distance-decay of predation from oyster reefs, the control reef was revisited over 24-25th July 2017.
Data exclusions	A handful of instances of lost data labels precluded the use of some data. All other data were used.
Reproducibility	The experiment has not been reproduced but the environmental context of the experiment (including MPB over the preceding 25 years in Echappe et al., 2018) has been exhaustively quantified.
Randomization	Of two reefs, one reef was kept as a control while oysters of the other were sacrificed. Sampling was randomised wherever possible, such as within cells of a regular spatial grid surrounding the oyster reefs, used to ensure wide coverage.
Blinding	Coordinates were blindly sampled by computer and GPS used to trace them exactly in the field.

Did the study involve field work? ☒ Yes ☐ No

Field work, collection and transport

Field conditions	Field visibility was good on low-tide sampling days.
Location	Two groups of oyster reefs within Bourgneuf Bay (47°01’32” N, 2°00’26” W), approximately 2 m above chart datum.
Access & import/export	No permits were necessary but local fishers and oyster farmers were conferred with about the plans well in advance.
Disturbance	Field workers were instructed to share pathways across the mud and used hovercraft or boats when moving heavy samples.

Reporting for specific materials, systems and methods

We require information from authors about some types of materials, experimental systems and methods used in many studies. Here, indicate whether each material, system or method listed is relevant to your study. If you are not sure if a list item applies to your research, read the appropriate section before selecting a response.

Materials & experimental systems

n/a	Involved in the study
☒	☐ Antibodies
☒	☐ Eukaryotic cell lines
☒	☐ Palaeontology and archaeology
☐	☒ Animals and other organisms
☒	☐ Human research participants
☒	☐ Clinical data
☒	☐ Dual use research of concern

Methods

n/a	Involved in the study
☒	☐ ChIP-seq
☒	☐ Flow cytometry
☒	☐ MRI-based neuroimaging

Animals and other organisms

Policy information about studies involving animals; ARRIVE guidelines recommended for reporting animal research

Laboratory animals	The study did not use laboratory animals.
Wild animals	All invertebrates and fish caught in the sampling were sorted from samples on site and transferred to containers for fixing with formalin. This was needed to reliably identify and measure biomass of species in the laboratory.
Field-collected samples	Field collected samples were fixed in formalin and stored at room temperature at the laboratory. They could then be handled by trained technicians during regular working hours.
Ethics oversight	No ethical approval was required because we handled no cephalopods and the only vertebrates were non-endangered fish species. These reefs were also wild populations of an invasive species, Crassostrea gigas, to north-west Europe.

Note that full information on the approval of the study protocol must also be provided in the manuscript.
